# Effects of lithium isotopes on sodium/lithium co-transport and calcium efflux through the sodium/calcium/lithium exchanger in mitochondria

**DOI:** 10.3389/fphys.2024.1354091

**Published:** 2024-04-09

**Authors:** Irina Bukhteeva, Fasih A. Rahman, Brian Kendall, Robin E. Duncan, Joe Quadrilatero, Evgeny V. Pavlov, Michel J. P. Gingras, Zoya Leonenko

**Affiliations:** ^1^ Department of Physics and Astronomy, University of Waterloo, Waterloo, ON, Canada; ^2^ Waterloo Institute for Nanotechnology, University of Waterloo, Waterloo, ON, Canada; ^3^ Department of Kinesiology & Health Sciences, University of Waterloo, Waterloo, ON, Canada; ^4^ Department of Earth and Environmental Sciences, University of Waterloo, Waterloo, ON, Canada; ^5^ Department of Molecular Pathobiology, New York University, New York, NY, United States; ^6^ Department of Biology, University of Waterloo, Waterloo, ON, Canada

**Keywords:** lithium isotopes fractionation, mitochondria, NCLX, Na/Ca/Li exchange, fluorescence, ICP-MS

## Abstract

The effects of lithium (Li) isotopes and their impact on biological processes have recently gained increased attention due to the significance of Li as a pharmacological agent and the potential that Li isotopic effects in neuroscience contexts may constitute a new example of quantum effects in biology. Previous studies have shown that the two Li isotopes, which differ in mass and nuclear spin, have unusual different effects *in vivo* and *in vitro* and, although some molecular targets for Li isotope fractionation have been proposed, it is not known whether those result in observable downstream neurophysiological effects. In this work we studied fluxes of Li^+^, sodium (Na^+^) and calcium (Ca^2+^) ions in the mitochondrial sodium/calcium/lithium exchanger (NCLX), the only transporter known with recognized specificity for Li^+^. We studied the effect of Li^+^ isotopes on Ca^2+^ efflux from heart mitochondria in comparison to natural Li^+^ and Na^+^ using Ca^2+^-induced fluorescence and investigated a possible Li isotope fractionation in mitochondria using inductively coupled plasma mass spectrometry (ICP-MS). Our fluorescence data indicate that Ca^2+^ efflux increases with higher concentrations of either Li^+^ or Na^+^. We found that the simultaneous presence of Li^+^ and Na^+^ increases Ca^2+^ efflux compared to Ca^2+^ efflux caused by the same concentration of Li^+^ alone. However, no differentiation in the Ca^2+^ efflux between the two Li^+^ isotopes was observed, either for Li^+^ alone or in mixtures of Li^+^ and Na^+^. Our ICP-MS data demonstrate that there is selectivity between Na^+^ and Li^+^ (greater Na^+^ than Li^+^ uptake) and, most interestingly, between the Li^+^ isotopes (greater ^6^Li^+^ than ^7^Li^+^ uptake) by the inner mitochondrial membrane. In summary, we observed no Li^+^ isotope differentiation for Ca^2+^ efflux in mitochondria via NCLX but found a Li^+^ isotope fractionation during Li^+^ uptake by mitochondria with NCLX active or blocked. Our results suggest that the transport of Li^+^ via NCLX is not the main pathway for Li^+^ isotope fractionation and that this differentiation does not affect Ca^2+^ efflux in mitochondria. Therefore, explaining the puzzling effects of Li^+^ isotopes observed in other contexts will require further investigation to identify the molecular targets for Li^+^ isotope differentiation.

## 1 Introduction

Lithium (Li), administered in the form of simple lithium carbonate or citrate salts, has been a forefront medication in the treatment of bipolar disorder for decades (Shorter, 2009). Despite its long-standing clinical use, the precise mechanism of action of Li, acting as a simple Li ion (Li^+^), in treating this disorder remains poorly understood (Kerr, Bjedov, and Sofola-Adesakin, 2018). From an entirely different perspective, Li, due to reported unusual isotope effects, has recently attracted a renewed attention from researchers (Fisher, 2015; Weingarten, Doraiswamy, and Fisher, 2016; Ettenberg et al., 2020; Deline et al., 2023; Livingstone et al., 2023). Li has two stable isotopes, ^6^Li and ^7^Li, with different atomic masses and nuclear spins. Specifically, ^6^Li has an atomic mass of 6.0151223 atomic mass units (amu) and a nuclear spin of 1, while ^7^Li has an atomic mass of 7.016004 amu and a nuclear spin of 3/2. Previous studies showed that Li^+^ isotopes have different effects on animal behaviour and activity (Lieberman, Alexander, and Stokes, 1979; Sechzer et al., 1986; Ettenberg et al., 2020), on electrical response in neuronal tissues (Esmaeilpour et al., 2023), on uptake by the cortex (Sherman, Munsell, and Wong, 1984), on animal lethality (Alexander et al., 1982), on mitochondria calcium (Ca^2+^) buffering capacity and on the properties of amorphous calcium phosphate aggregates (ACP) *in vitro* (Deline et al., 2023; Fisher, 2023). A recent study reported that the sodium (Na^+^)/proton (H^+^) exchangers in fibroblast cells fractionate Li^+^ isotopes (Poet et al., 2023). On the other hand, other studies have found no difference in biochemical or cellular processes (Parthasarathy and Eisenberg Jr, 1984; Livingstone et al., 2023).

While certain studies (Lieberman, Alexander, and Stokes, 1979; Alexander et al., 1982; Sherman, Munsell, and Wong, 1984; Ettenberg et al., 2020; Deline et al., 2023) highlight some difference in Li^+^ isotope effects, a subset of the research results on lithium isotope effects in biological systems stands out in having revealed significant and contrasting impacts by each Li^+^ isotope. For example, in the study by Sechzer et al. (1986) the two Li^+^ isotopes were reported to provoke opposite maternal behaviour while, in a very recent study, Esmaeilpour et al. (2023) found a large and opposite effect on the electrical response in neuronal rat hippocampal tissues. The overarching question that these studies raise is: what are the possible molecular targets that may be responsible for such differentiation in Li^+^ isotope effects that may lead to such substantial and opposing outcomes? It is in this vein that we investigated whether the two Li^+^ isotopes may affect differently the Li^+^-induced Ca^2+^ efflux in heart mitochondria.

The specific mechanisms and the level at which Li^+^ isotopes act to manifest their different effects are unclear. The possibility of different Li^+^ isotope effects in neuroscience contexts is both puzzling and intriguing and has led to the formulation of new theoretical hypotheses invoking various quantum properties of the Li isotopes, such as its nuclear spin, that may determine their relative specific activity (Fisher, 2015; Weingarten, Doraiswamy, and Fisher, 2016; Zadeh-Haghighi and Simon, 2021). Recent work (Poet et al., 2023) reported Li^+^ isotope fractionation via the Na^+^/H^+^ exchanger in fibroblast cells, rationalizing the phenomenon in terms of a kinetic isotope effect involving the mass difference between the two isotopes. From a broader perspective, were neurophysiological Li^+^ isotope effects to be vindicated, it would constitute a new example of quantum effects arising in biology—i.e., within the field of quantum biology (Abbott, Davies, and Pati, 2008; Mohseni et al., 2014; Marais et al., 2018).

**FIGURE 1:**
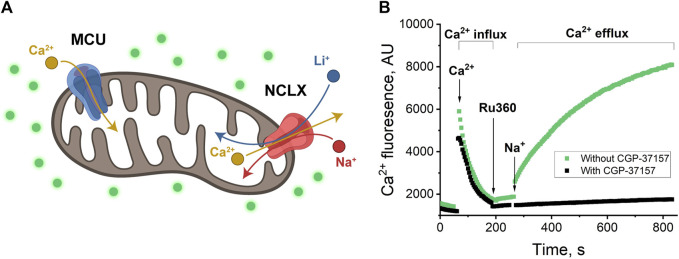
**(A)** Schematics of mitochondrion with MCU and NCLX channels. MCU passes Ca^2+^ inside the mitochondrion (Ca^2+^ influx); NCLX passes Na^+^ or Li^+^ inside the mitochondrion in exchange for Ca^2+^ transport outside (Ca^2+^ efflux) (Rossi, Pizzo, and Filadi, 2019; Walters and Usachev, 2023). **(B)** Ca^2+^-induced fluorescence as a measure of Ca^2+^ influx through MCU and Ca^2+^ efflux through NCLX in exchange for Na^+^. Mitochondria were added to the buffer with/without CGP-37157 and were allowed to equilibrate. Then, 20 μM of CaCl_2_ was added and allowed to equilibrate, which resulted in a sharp increase of fluorescence in the first portion of the plot (around 50–60 s), followed by a fluorescence decrease due to Ca^2+^ uptake by mitochondria via MCU channel, from 100 to 200 s (Ca^2+^ influx). 10 μM Ru360 was added to inhibit MCU and, approximately 60 s after, 20 mM of NaCl was added to initiate Na^+^ exchange for Ca^2+^ via NCLX. The increase in fluorescence (green) is visible after 260 s when NCLX is functional and almost no increase (black) when NCLX is inhibited by CGP-37157. Panel **(B)** shows a representative trace of mitochondrial extract from one animal with one technical replicate.

Although Li has long had clinical applications and is present in various natural sources, the full extent of its molecular targets in organisms has not been fully identified. While many Li^+^ targets have been recognized and investigated, sometimes with consideration of Li^+^ isotope-dependent processes (Parthasarathy and Eisenberg Jr, 1984; Parthasarathy et al., 1992; Livingstone et al., 2023), a complete knowledge and understanding of which ones are crucial for their neurological potency is still lacking. One of the primary pathways for Li^+^ entry into cells is through sodium (Na^+^) channels, where Li^+^ can compete with Na^+^ for intra-channel binding sites (Richelson, 1977; Thomsen and Shirley, 2006), thus indicating that Li^+^ and Na^+^ share affinities for these specific locations. It is worth noting that Li^+^ can also pass through potassium (K^+^) channels and interfere with K^+^ transport (Thompson et al., 2009). Furthermore, Li^+^ can enter Ca^2+^ channels and inhibit Ca^2+^ flux (Kuo and Hess, 1993). In addition to ion channels, Li^+^ can also be transported by and regulate the activity of the mitochondrial sodium/calcium/lithium exchanger (NCLX), which was identified as not only a Na^+^ to Ca^2+^ exchanger, but also as a Li^+^ to Ca^2+^ exchanger (Boyman et al., 2013). Mitochondrial Ca^2+^ transport regulates cell bioenergetics, Ca^2+^ signaling, and cell death. The influx and accumulation of Ca^2+^ in mitochondria is facilitated by the mitochondrial Ca^2+^ uniporter (MCU), while Ca^2+^ is extruded through the mitochondrial NCLX and H^+^/Ca^2+^ exchangers (Figure 1A). It was shown that liver has significantly higher NCLX expression levels than brain or heart mitochondria (Rysted et al., 2021). However, these last authors suggested that NCLX is responsible for Ca^2+^ extrusion from the mitochondria in the brain and heart, while playing only a minimal role, if any, in liver mitochondria. The NCLX facilitates the exchange of Na^+^ or Li^+^ for Ca^2+^ across the mitochondrial *inner membrane,* initiating Ca^2+^ efflux. Ca^2+^ is a crucial signaling ion in the brain, playing a pivotal role in various neuronal processes. It serves as a second messenger in intracellular signaling cascades and participates in neurotransmitter release, synaptic plasticity, and neuronal excitability. The precise regulation of Ca^2+^ levels is essential for proper neuronal function and communication. As Li^+^ is known to compete with other ions and influence calcium signaling (Dubovsky and Franks, 1983; Glen, 1985; Helmeste and Tang, 1998; Bosche et al., 2013; Harrison et al., 2021), we wanted to test whether Li^+^ isotopes differently affect Na^+^/Ca^2+^/Li^+^ exchange in NCLX.

The fact that NCLX, an ion exchanger of biological relevance, has been given a dedicated acronym that reflects its established ability to transport Li^+^ naturally motivates one to consider it as a natural target to explore in terms of its response to the two Li^+^ isotopes. In this vein, our study aimed to uncover the effects of different Li^+^ isotopes on the NCLX exchanger in mouse heart mitochondria. Specifically, we measured the rates of Ca^2+^ efflux associated with NCLX using Ca^2+^-induced fluorescence and investigated a possible Li^+^ isotope fractionation within the mitochondrial matrix using inductively coupled plasma mass spectrometry (ICP-MS). We selected heart mitochondria as our model for several reasons. Firstly, heart mitochondria are the most extensively studied among mitochondria from various tissues. Secondly, the extraction of heart mitochondria is rather straightforward, providing a higher yield for multiple experiments and minimizing animal usage, in line with animal ethics guidelines. Finally, heart mitochondria bear significant similarities to brain mitochondria and, perhaps most importantly, are an exceedingly well-established model for studying NCLX and mitochondrial processes (Rysted et al., 2021). Thus the heart mitochondrion constitutes an excellent test candidate for a first exploration of the proposal that NCLX transport may be differently affected by the two Li^+^ isotopes.

## 2 Materials and methods

### 2.1 Chemicals

Li salts, ^6^LiCl (95% ^6^Li) and ^7^LiCl (99% ^7^Li), as well as natural LiCl salt (with natural abundance - ^6^Li at 7.49% and ^7^Li at 92.51%) were purchased from Sigma-Aldrich. TMRE (T669), Calcium Green 5-N (C3737) and trace-metal grade HNO_3_ were purchased from Fisher Scientific. Percoll was purchased from Cedarlane. All other reagents were purchased from Sigma-Aldrich.

### 2.2 Mitochondria isolation

All procedures using mice were performed at the University of Waterloo (UW) according to animal use protocols that received ethics approval by the UW Animal Care Committee (i.e., AUPP#43325, approved 3 Jun 2021, and AUPP#44128, approved 11 March 2022), and complied with Canadian Council on Animal Care guidelines. Mice were housed in a temperature- and humidity-controlled environment in same-sex groups under a 12:12-h light/dark cycle, with free access to standard rodent chow (Teklad 22/5 Rodent diet from Envigo, Haslett, MI, USA) and water. Enrichment materials were provided. Cardiac mitochondria were isolated from male and female adult (i.e., 9–12 weeks old) C57BL/6J mice using a standard protocol (Elustondo et al., 2015; Rysted et al., 2021). In brief, whole hearts were harvested immediately after euthanasia by cervical dislocation, and the tissue was minced, washed with 1 × PBS containing 10 mM EDTA buffer (pH = 7.4), and digested with trypsin for 15 min, then homogenized in ice-chilled mitochondria isolation buffer (100 mM KCl, 50 mM MOPS, 5 mM MgSO_4_-7H_2_O, 1 mM EGTA, 0.1% BSA, pH 7.4 with KOH). The homogenate was centrifuged at 500 × *g* for 5 min, and then the supernatant was centrifuged at 10,000 × *g* for 10 min. The pellet was then resuspended in an isolation buffer, and layered on top of a Percoll gradient that was centrifuged at 7000 × *g* for 5 min. The final pellet was resuspended in sucrose wash buffer (250 mM sucrose, 10 mM HEPES, 0.1% BSA, pH 7.2), and the protein content of the sample was determined using the BCA method (Zhou et al., 2023). All pH adjustments were made with KOH and HCl to exclude the addition of extra Na^+^, which affect experimental results.

### 2.3 Ca^2+^ fluorescence experiments

Mitochondrial Ca^2+^ flux was monitored using the low-affinity fluorescent label Calcium Green 5N, as a Ca^2+^ indicator (Kd∼14 µM) and measuring changes in the extramitochondrial Ca^2+^ concentration. Specifically, mitochondria were suspended in a respiration buffer (5 mM succinate, 300 mM sucrose, 20 mM TRIS, 0.2 mM KH_2_PO_4_, 1 µM Ca Green, 0.8 µM Rotenone, 5 µM CSA) in a 96 well, black plate. Ca Green 5N was excited at 500 nm and fluorescence collected at 541 nm using a Cytation5 Reader. For a standard experiment, mitochondria were added to the respiration buffer and equilibrated. Then, they were loaded with Ca^2+^ (20 µM), to enable uptake of Ca^2+^ by mitochondria via mitochondrial calcium uniporter (MCU), followed by the addition of the MCU inhibitor Ru360 (0.1 µM) to prevent further mitochondrial Ca^2+^ transport via MCU, thereby enabling the study of mitochondrial Ca^2+^ efflux via NCLX only. To probe for the role of NCLX, 20 mM of NaCl or 20 mM LiCl was added to mitochondria in the continuous presence of Ru360. For the inhibited NCLX study, CGP-37157 (30 µM) was added to the respiration buffer.

The rate of Ca^2+^ efflux was quantified as Ca^2+^ fluorescence per minute per mg of mitochondrial protein during the first minute of any given condition.

### 2.4 Inductively coupled plasma mass spectrometry (ICP-MS)

For the ICP-MS experiments, mitochondria were added to the respiration buffer and equilibrated. They were then loaded with Ca^2+^ (20 µM), followed by the addition of the MCU inhibitor Ru360 (0.1 µM) to prevent mitochondrial Ca^2+^ uptake. To determine the difference in Li^+^ isotopes and ^nat^Li^+^ versus Na^+^ passages, mitochondria were treated with a 20 mM Li^+^ isotope mix (10 mM ^6^Li^+^ and 10 mM ^7^Li^+^), or a 15 mM Na^+^/Li^+^ mix (5 mM Na^+^ and 10 mM ^nat^Li^+^) in the continuous presence of Ru360. To scrutinize the passage of Li^+^ in NCLX, in some of the experiments, NCLX was blocked by the addition of CGP-37157 (30 μM) to the respiration buffer. After 10 min, the mitochondria solutions were centrifuged (7,000 × *g* for 5 min), then supernatants and mitochondria pellets were separated and used for further analysis. The mitochondria pellets were triple-washed with resuspension to remove trapped Li^+^ or Na^+^ between mitochondria and in the intermembrane mitochondrial space with respiration buffer and inhibitors (Ru360 and CGP-37157). To confirm the effective elimination of Li^+^ or Na^+^ trapped in mitochondria pellets, the supernatant washes obtained after centrifugation (in the absence of mitochondrial samples) were subjected to ICP-MS analysis. The results revealed a rapid reduction in ion concentrations, meaning that trapped ions were successfully removed. The concentration of Li^+^ in the third wash was measured to be ∼10^4^ times lower than in the supernatant, approaching a zero-value baseline, while the concentration of the Na^+^ also decreased, albeit not as drastic as for Li^+^, returning to the buffer nonzero base level (data not presented).

Next, purified mitochondria pellets and supernatants were analyzed with ICP-MS for Li^+^ and Na^+^ contents. The samples were treated as previously described (Deline et al., 2023). Briefly, each sample was digested in 1 mL concentrated (67%–70%, Fisher Scientific trace metal grade) HNO_3_ and 1 mL 30% H_2_O_2_ (Millipore Suprapur) for 1 h at 110°C to eliminate organic matter. Samples were evaporated to dryness and then diluted in 3 mL of 2% trace metal grade HNO_3_. The concentrations of ^6^Li, ^7^Li, and Na were determined using an Agilent 8800 triple quadrupole inductively coupled plasma mass spectrometer (QQQ-ICP-MS). Each sample solution was measured ten times, with each measurement comprising 1000 sweeps of the mass spectrum and 2-second total integration times for each Li isotope. Instrument drift was corrected using scandium (Sc) as the internal element standard. Instrumental accuracy was verified using multiple United States Geological Survey (USGS) and NIST SRM 1643f water standards and ^7^Li, ^6^Li, ^nat^Li, and mixed Li (∼47%/53% mix of ^6^Li and ^7^Li) control standards.

### 2.5 Statistical analysis

All Ca^2+^ fluorescence data were collected from a minimum of 3 biological replicates (defined as mitochondria extracts from a whole individual mouse heart) and 2 technical replicates (defined as multiple experiments from a given mouse heart). Data were analyzed by one-way ANOVA with Bonferroni’s *post hoc* test (for comparing >2 groups of data). Means ± SEM are plotted throughout, and the following significance levels are reported: n.s. (*p* > 0.05), *p* < 0.05, *p* < 0.01, *p* < 0.001, and *p* < 0.0001.

ICP-MS data were collected from 2 to 3 biological replicates (defined as mitochondria extracts from a whole individual mouse heart). Measurements resulting in less than 1 ppb Li were discarded since they did not yield reproducible results as the accuracy of standard Li isotope ratios degrades at low Li concentrations.

## 3 Results

Recapping what was stated in the Introduction, mitochondria are known for storing Ca^2+^ and releasing it by exchanging it with Na^+^ or Li^+^ through NCLX. Two naturally occurring isotopes, ^6^Li and ^7^Li, have been observed to produce distinct effects on animal behaviour and activity (Lieberman, Alexander, and Stokes, 1979; Sechzer et al., 1986; Ettenberg et al., 2020), on electrical response in neuronal tissues (Esmaeilpour et al., 2023), and on mitochondria calcium buffering capacity (Deline et al., 2023). Most recently, the two Li^+^ isotopes have been found to differently affect the formation and properties of ACP *in vitro* (Fisher, 2023). Given the established role of Ca^2+^ in neuronal processes, we hypothesize that Li^+^ isotopes may have varying effects on Ca^2+^ release in mitochondria, ultimately leading to different downstream outcomes. Henceforth, for compactness, we refer to lithium salt samples with the natural isotopic abundance (^6^Li at 7.49% and ^7^Li at 92.51%) simply as “natural lithium.”

### 3.1 Ca^2+^ influx, efflux and NCLX activity for Na^+^/Ca^2+^ exchange measured with Ca^2+^-induced fluorescence

To measure Ca^2+^ efflux, we used the Ca Green-5N fluorescence label, which selectively fluoresces when bound to free Ca^2+^. Ca Green-5N was added to the buffer outside the mitochondria and, as it is unable to permeate into mitochondria, it provides a means to quantify the Ca^2+^ efflux by measuring fluorescence intensity. The fluorescence signal increases with elevated levels of free Ca^2+^ outside the mitochondria.

To establish a control experiment (Figure 1B) the fluorescence was measured to follow both Ca^2+^ influx and efflux: the mitochondria were introduced to the respiration buffer and allowed to equilibrate (Figure 1B, pre-time = 0). Subsequently, they were loaded with 20 µM Ca^2+^ (Figure 1B, indicated by the arrow at time = 100 s), followed by the addition of the MCU inhibitor Ru360 (Figure 1B, indicated by the arrow at time = 200 s) at 0.1 µM to prevent mitochondrial Ca^2+^ uptake. This design enabled the investigation of mitochondrial Ca^2+^ efflux.

Next, 20 mM of the selected salt (NaCl, in this example, indicated by the arrow at time = 280 s) was introduced to the mitochondria (Figure 1B, green curve). An increase in fluorescence during the time period spanning 280–800 s indicates Ca^2+^ efflux.

To inhibit the transport of ions through the NCLX channel, 30 µM CGP-37157 (NCLX inhibitor) was introduced to the respiration buffer. With the presence of CGP-37157, we observe a stable background fluorescence signal that just slightly increases overtime, indicating the essentially complete absence of Ca^2+^ release (Figure 1B, black curve).

With this experiment, we established our control and confirmed that the process of Ca^2+^ influx and efflux and Na^+^ to Ca^2+^ exchange via NCLX is operating in agreement with previously reported (Rysted et al., 2021) protocols in mice heart mitochondria at similar conditions.

These protocols were then applied in the following experiments to monitor Ca^2+^ efflux as a result of exchange between Ca^2+^ and Li^+^, as well as between Ca^2+^ and each of the two Li^+^ isotopes.

#### 3.1.1 NCLX activity and Ca^2+^ exchange for Na^+^ and Li^+^ (natural lithium)

To assess NCLX activity, we examined Ca^2+^-induced fluorescence in the presence of different salts: NaCl, KCl, and natural LiCl (^nat^LiCl) — both with and without CGP-37157 inhibitor. We anticipated that Na^+^ would induce a higher Ca^2+^ efflux than Li^+^ (Rysted et al., 2021), while K^+^ was not expected to elicit any Ca^2+^ release and was employed as a negative control. Following the control experimental methodology developed with Na^+^, described in Section 3.1 and Figure 1B, and through titration experiments with Na^+^ and Li^+^, we determined that a salt concentration of 20 mM yielded maximum Ca^2+^ efflux and was consequently utilized in all experiments (refer to Supplementary Figures S1A–D). While all procedures with Na^+^, K^+^ and Li^+^ were followed exactly as in the control experiment shown in Figure 1B, only the Ca^2+^ efflux portion of the experiments is presented and discussed in the plots below.

**FIGURE 2:**
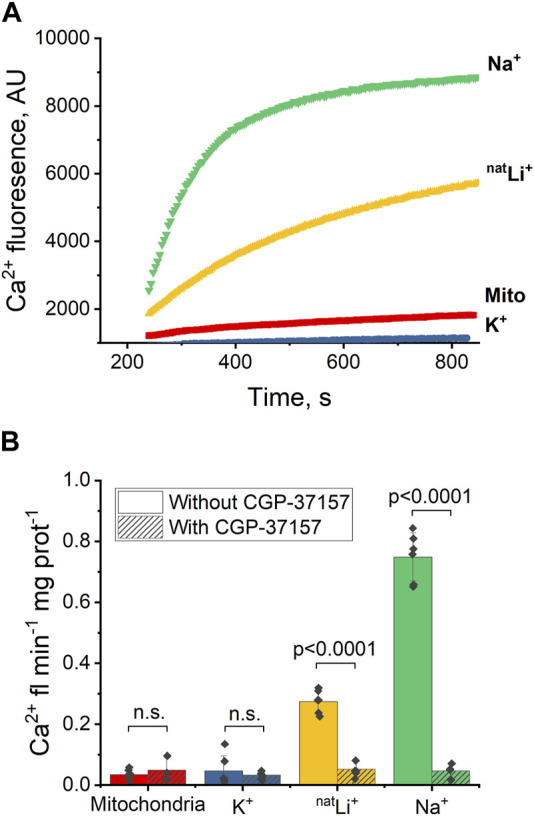
Ca^2+^-induced fluorescence as a probe of NCLX activity in the presence of Na^+^, Li^+^ and K^+^. **(A)** Representative Ca^2+^ efflux measures from isolated heart mitochondria during a 10 min period where NCLX is functional in the presence of 20 mM of Na^+^, ^nat^Li^+^, K^+^, or mitochondria alone. **(B)** Quantification of the maximal rates of mitochondrial Ca^2+^ efflux induced by Ru360 alone, 20 mM Na^+^, ^nat^Li^+^, or K^+^ in the continuous presence of Ru360 in case of functional or inhibited NCLX.

Upon introducing 20 mM NaCl (Figure 2A, green) or LiCl (Figure 2A, yellow) to the extramitochondrial solution, a pronounced increase in fluorescence was observed, signifying an induced Ca^2+^ efflux from the mitochondria, and thus indicating a successful exchange of Ca^2+^ for Na^+^ or Li^+^ via NCLX. To quantify and compare the Ca^2+^ effluxes, we utilized the Ca^2+^ efflux rate, measured as Ca^2+^-induced fluorescence per minute per mg of mitochondrial protein during the initial minute of any given condition (maximal slope). The Ca^2+^ efflux rate triggered by Li^+^ was significantly slower (*p* < 0.0001) than that induced by Na^+^ (Figure 2B, yellow and green, respectively). Notably, this Na^+^/Li^+^-induced Ca^2+^ efflux was inhibited by the application of the NCLX inhibitor CGP-37157 (Figure 2B, yellow and green, dashed). Conversely, the introduction of 20 mM KCl (Figures 2A, B, blue) did not stimulate mitochondrial Ca^2+^ efflux, as evidenced by the absence of increased fluorescence.

These results indicate that Na^+^ and Li^+^ trigger Ca^2+^ efflux via NCLX, and that Ca^2+^ efflux in response to Na^+^ is significantly higher than in response to Li^+^. CGP-37157 efficiently inhibits Ca^2+^ efflux triggered by the presence of Li^+^ or Na^+^. As expected, K^+^ do not exchange for Ca^2+^ via NCLX.

Next, we explored Ca^2+^ efflux stimulated by the simultaneous presence of Na^+^ and Li^+^. Previous studies have proposed distinct binding sites for Na^+^ and Li^+^ (Giladi et al., 2022), so we sought to examine how the simultaneous presence of Na^+^ and Li^+^ would influence Ca^2+^ efflux.

**FIGURE 3:**
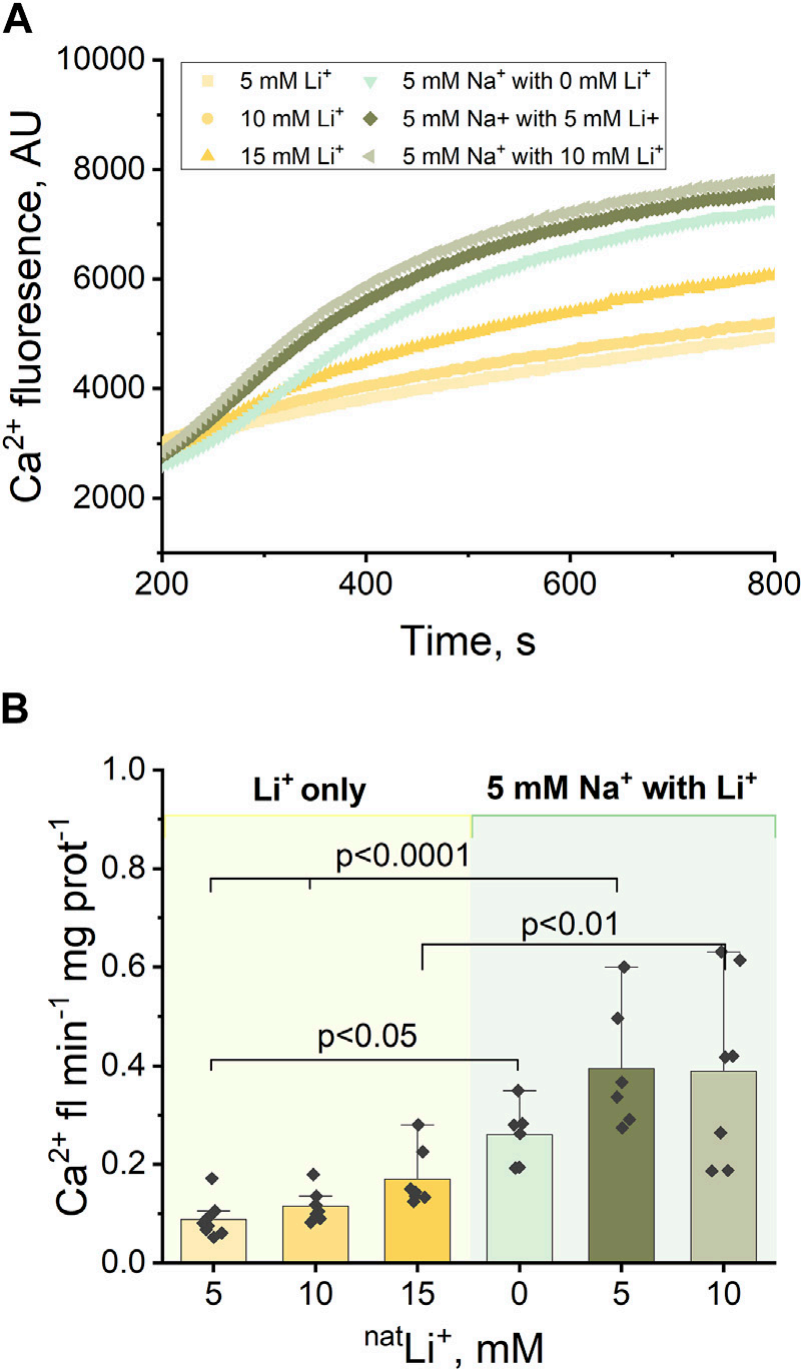
Ca^2+^-induced fluorescence as a measure of Ca^2+^efflux in response to 5 mM Na^+^ and different concentrations of ^nat^Li^+^ (5, 10 and 15 mM). **(A)** Representative Ca^2+^ efflux measurements from isolated heart mitochondria during a 10 min period where NCLX is functional in the presence of 5 mM Na^+^ and different concentrations of ^nat^Li^+^. **(B)** Quantification of the maximal rates of mitochondrial Ca^2+^ efflux induced by 5 mM Na^+^ and different concentrations of ^nat^Li^+^ in the continuous presence of Ru360.

The experimental protocol outlined in Section 3.1 (Figure 1B), was adapted to incorporate varying concentrations of LiCl (5 and 10 mM) alongside 5 mM NaCl (Figures 3A, B). The results indicate that the Ca^2+^ efflux rate triggered by the simultaneous presence of Na^+^ and Li^+^ is significantly higher than the Ca^2+^ efflux rate triggered by Li^+^ alone: 5 mM Na^+^ and 10 mM Li^+^ results in 0.39 Ca^2+^ fl min^−1^ mg prot^-1^ compared to 0.17 Ca^2+^ fl min^−1^ mg prot^−1^ triggered by 15 mM Li^+^ (*p* < 0.01, Figure 3B; Supplementary Figures S1A, B); 5 mM Na^+^ and 5 mM Li^+^ results in 0.39 Ca^2+^ fl min^−1^ mg prot^−1^ compared to 0.12 Ca^2+^ fl min^−1^ mg prot^−1^ triggered by 10 mM Li^+^ (*p* < 0.001, Figure 3B). However, there is no statistically significant difference (*p* > 0.5) between the Ca^2+^ efflux rate triggered by the simultaneous presence of Na^+^ and Li^+^ and the Ca^2+^ efflux rate triggered by Na^+^ alone: 5 mM Na^+^ and 5 mM Li^+^ results in 0.39 Ca^2+^ fl min^−1^ mg prot^-1^ (Figure 3B) compared to 0.33 Ca^2+^ fl min^−1^ mg prot^−1^ triggered by 10 mM Na^+^ (Supplementary Figure S1B).

#### 3.1.2 NCLX activity in the presence of each Li^+^ isotope

**FIGURE 4:**
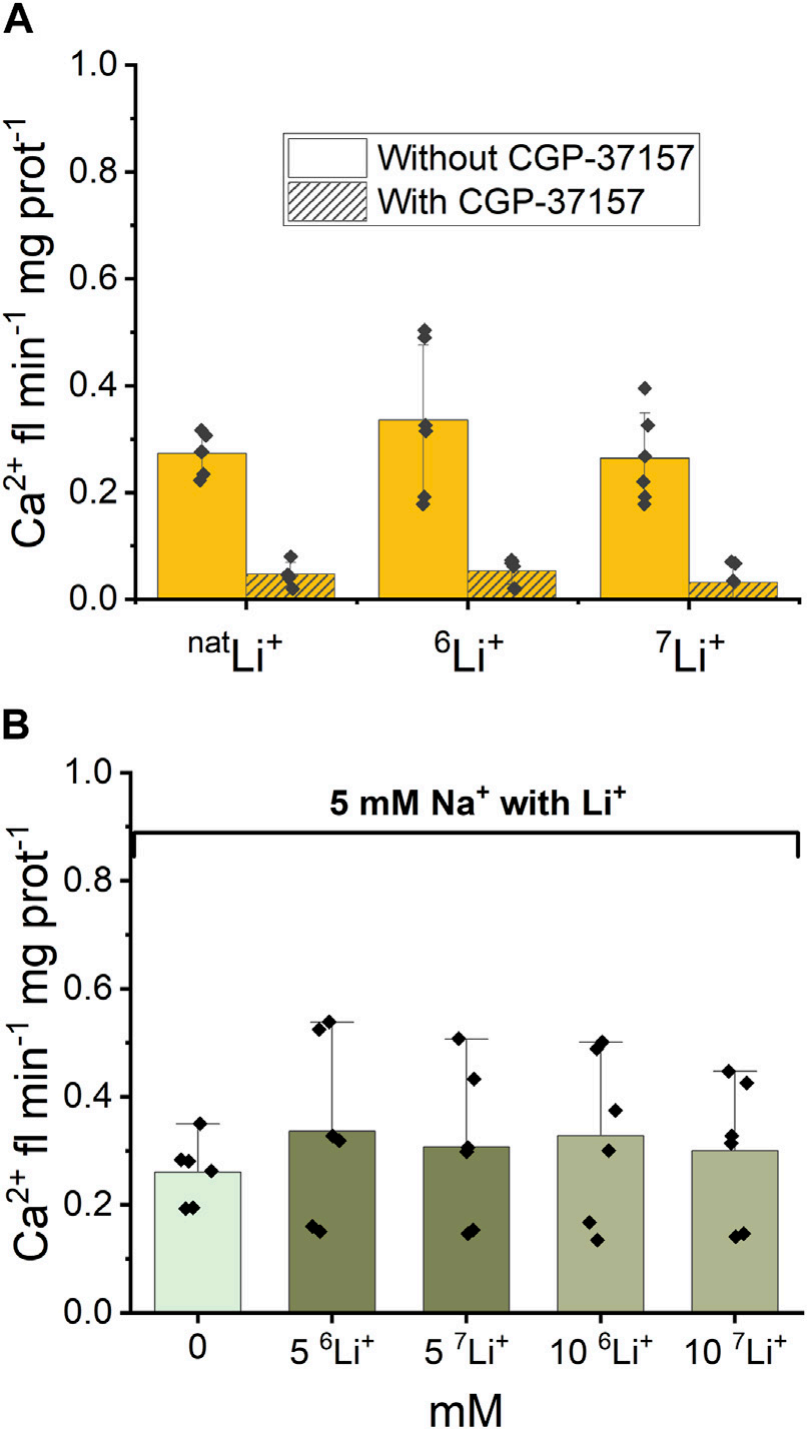
Ca^2+^-induced fluorescence as a measure of Ca^2+^ efflux in response to Li^+^ isotope presence. **(A)** Quantification of the maximal rates of mitochondrial Ca^2+^ efflux induced by 20 mM ^nat^Li^+^, ^6^Li^+^ and ^7^Li^+^ in the continuous presence of Ru360 in the case of functional and inhibited NCLX. **(B)** Quantification of the maximal rates of mitochondrial Ca^2+^ efflux induced by 5 mM Na^+^ and different concentrations of ^nat^Li^+^, ^6^Li^+^ and ^7^Li^+^ in the continuous presence of Ru360 in the case of functional (i.e., uninhibited) NCLX.

Following the examination of NCLX properties, parallel studies were conducted using ^6^LiCl and ^7^LiCl salts. The introduction of 20 mM ^6^LiCl or ^7^LiCl to the extra-mitochondrial solution resulted in an observable increase in Ca^2+^ fluorescence, indicating an enhancement in Ca^2+^ efflux from the mitochondria. Figure 4A illustrates that mitochondrial Ca^2+^ efflux rates triggered by either Li^+^ isotope were comparable to the Ca^2+^ efflux induced by ^nat^Li salts. To test the specific role of NCLX in this process the NCLX exchanger was either left functional (without CGP-37157) or inhibited by CGP-37157 (Figure 4A, dashed).

Furthermore, as depicted in Figure 4B, there was no statistically significant difference (*p* > 0.5) observed in Ca^2+^ efflux rates between the simultaneous presence of 5 mM Na^+^ and 0–10 mM natural Li^+^ or either Li^+^ isotope.

Thus, fluorescence data show that there is no difference in Ca^2+^ efflux rate triggered by ^nat^Li^+^, ^6^Li^+^ or ^7^Li^+^ when Li salts were applied alone or in combination with 5 mM Na^+^. The CGP-37157 inhibitor efficiently inhibits Ca^2+^ efflux triggered by the presence of ^nat^Li^+^, ^6^Li^+^ or ^7^Li^+^, irrespective of which isotope is present.

### 3.2 Uptake of ^nat^Li^+^ vs. Na^+^ and ^6^Li^+^ vs. ^7^Li^+^ into mitochondria

We utilized ICP-MS to investigate the uptake level of Li^+^ and Na^+^ across the mitochondrial inner membrane—in the matrix. This analytical approach allows for the precise quantification of element concentrations and ratios within a sample. We hypothesized that there might be a different uptake of Na^+^ and Li^+^, and thus the ratio of Li^+^/Na^+^ inside the mitochondrion matrix would deviate from the original ratio in the extra-mitochondrial buffer.

**FIGURE 5:**
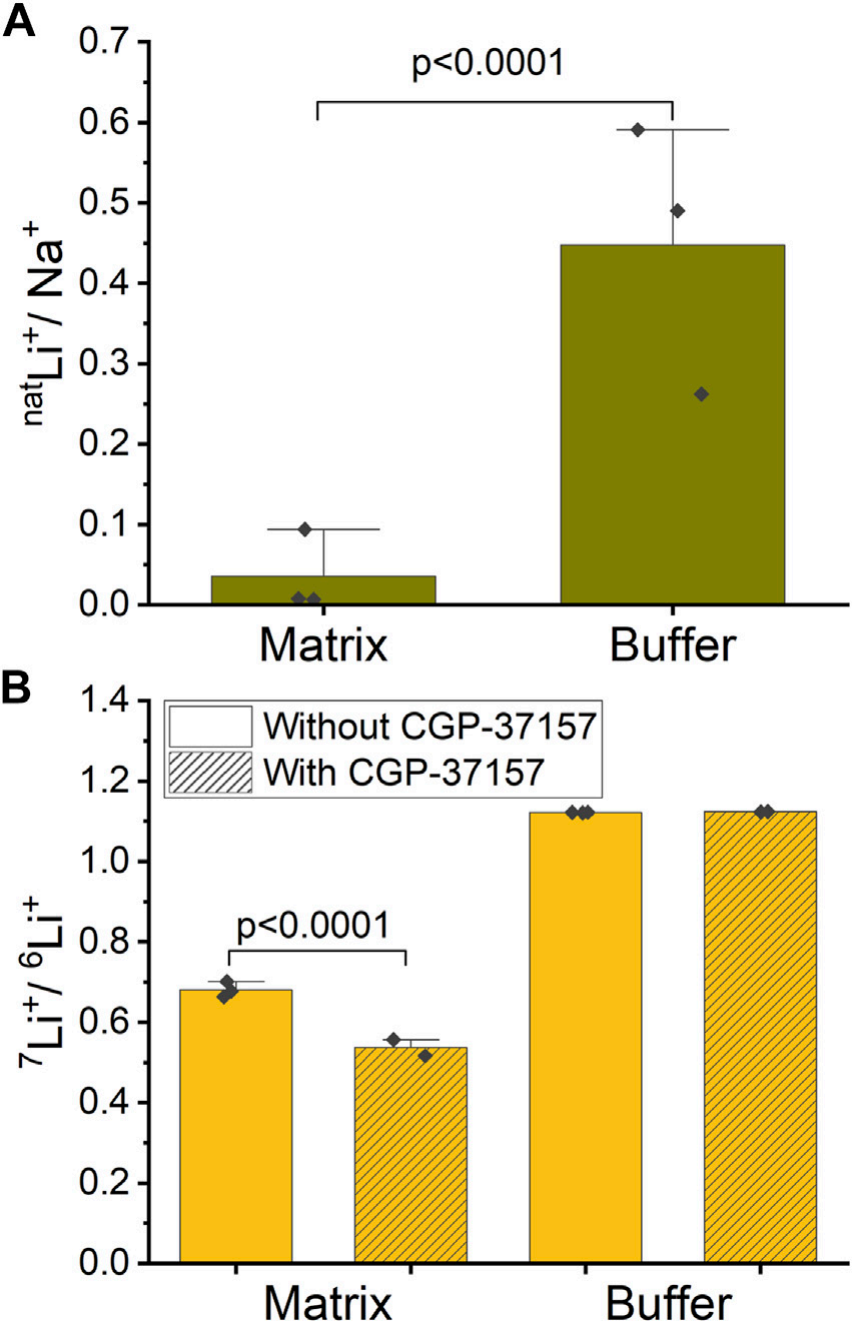
Ion ratios inside and outside of mitochondria measured by ICP-MS. **(A)** Mitochondria were treated with a mixture of ^nat^Li^+^ and Na^+^. The graph shows the ratio of ^nat^Li^+^/Na^+^ inside (mitochondrial matrix) and outside (buffer) of mitochondria in the case of functional NCLX. **(B)** Mitochondria were treated with an approximate 47%/53% solution of ^6^Li^+^/^7^Li^+^. The graph shows the ratio of ^7^Li^+^/^6^Li^+^ inside (matrix) and outside (buffer) of mitochondria with and without NCLX inhibition.

Mitochondria were exposed to a respiration buffer containing 5 mM NaCl and 10 mM ^nat^LiCl for 10 min, enabling NCLX to uptake Na^+^ and ^nat^Li^+^ by exchanging for Ca^2+^ and thus releasing it. Subsequently, samples underwent centrifugation, and both the buffer and washed pellet (matrix) were collected for analysis of each ion content ratio inside and outside mitochondria. To quantify the relative uptake of ^nat^Li^+^ versus Na^+^, we define a ^nat^Li^+^/Na^+^ quantity. We calculated that ^nat^Li^+^/Na^+^ = 0.036 ± 0.050 in matrix compared to ^nat^Li^+^/Na^+^ = 0.448 ± 0.159 in buffer (SD, *n* = 3, Figure 5A; Supplementary Table S1). These results suggest that mitochondria preferentially uptake Na^+^ over ^nat^Li^+^.

In a similar experiment, mitochondria were exposed to a respiration buffer containing 20 mM LiCl with close to equimolar isotope abundance (47% ^6^Li and 53% ^7^Li), as determined by ICP-MS. These solutions were applied to mitochondria, where NCLX was either inhibited by CGP-37157 or not. To quantify the Li^+^ isotope fractionation between the mitochondrion matrix and buffer, we calculated ^7^Li^+^/^6^Li^+^. For the functional NCLX, we determined that ^7^Li^+^/^6^Li^+^ = 1.122 ± 0.001 (SD, *n* = 3) in buffer and ^7^Li^+^/^6^Li^+^ = 0.681 ± 0.019 (SD, *n* = 3) in mitochondrial matrix. For the inhibited NCLX, ^7^Li^+^/^6^Li^+^ = 1.124 ± 0.001 (SD, *n* = 2) in buffer and ^7^Li^+^/^6^Li^+^ = 0.537 ± 0.029 (SD, *n* = 2) in mitochondrial matrix (Figure 5B; Supplementary Table S2). These results suggest that mitochondria preferentially uptake ^6^Li^+^ over ^7^Li^+^ with or without the presence of CGP-37157, with ^6^Li^+^ uptake more strongly favored in the presence of CGP-37157 blocking NCLX.

## 4 Discussion

Lithium (Li), administered in the form of carbonate or citrate salts, has long been used to treat bipolar disorder (Shorter, 2009), although its exact mechanism of action remains unclear (Kerr, Bjedov, and Sofola-Adesakin, 2018). Li has two stable isotopes, ^6^Li and ^7^Li, with varying effects on animal behaviour (Lieberman, Alexander, and Stokes, 1979; Sechzer et al., 1986; Ettenberg et al., 2020), electrical response in neuronal tissues (Esmaeilpour et al., 2023), mitochondrial Ca^2+^ buffering capacity (Deline et al., 2023), and the properties of amorphous calcium phosphate (ACP) clusters and their formation (Deline et al., 2023; Fisher, 2023). A recent study also reported that the Na^+^/H^+^ exchangers in fibroblast cells fractionate Li^+^ isotopes (Poet et al., 2023). The possibility of strong isotopic effects in neuroscience is highly unexpected. Were such isotopic differences to be rigorously demonstrated experimentally, those would have to be assigned to one, or both, of the two distinct properties of the two isotopes: their different mass and their different nuclear spin (note that nuclear magnetic dipole and electric quadrupole moments of the two isotopes also differ). Naively, on the basis of conventional atomic physics, and thus chemistry and biochemistry, those nuclear properties would have, evolutionary speaking, already been “folded in” the rather small differences each isotope would cause in terms of molecular bonding and ion channel transport when considering experiments with a natural abundance of the two Li isotopes. As such, any large experimental differences between the two Li isotopes would suggest that heretofore underappreciated significant quantum effects may be at play in Li’s neurological activity (Fisher, 2015; Weingarten, Doraiswamy, and Fisher, 2016; Ettenberg et al., 2020; Zadeh-Haghighi and Simon, 2021; Deline et al., 2023; Livingstone et al., 2023). Our own recent work (Esmaeilpour et al., 2023) demonstrates that a large and opposite effect of Li^+^ isotopes is observed in electrical activity (evoked field postsynaptic potential measurements) of animal brain slices, indicating that fast electrochemical processes associated with ion channels or synaptic transmission may be involved. The works by Deline et al. (2023) and Fisher (2023) indicate that the two Li^+^ isotopes differentially affect the properties of ACP and mitochondrial calcium buffering capacity, which suggests that downstream Ca^2+^ signaling could be affected by such. Processes of Ca^2+^ aggregation and storage in mitochondria are important in Ca^2+^ regulation and its dysregulation has been linked to various neurological disorders (Khacho, Harris, and Slack, 2019; Calvo-Rodriguez et al., 2020; Cascella and Cecchi, 2021).

Beyond Li, results from experiments considering isotopes of other elements have recently been found to challenge the traditional view that the different isotopes of a given element should minimally impact biology –– the case of proton (H) versus deuterium (D) is a well-known counterexample finding its origin in the large kinetic isotope effect driven by the 100% mass difference between H and D (Di Martino, Maxwell, and Pirali, 2023). Stable oxygen isotope fractionation in chiral environments has been reported (Vardi et al., 2023). Intriguingly, the efficacy of xenon-induced general anesthesia has recently been reported to exhibit a strong dependence on the xenon isotope considered (Li et al., 2018). Integrating these recent observations along with the aforementioned reports of Li isotope effects in a number of experiments of neurological context emphasizes that various isotopes of a given element, not just Li, may have distinct effects. These observations could pave the way for uncovering unexpected quantum effects emerging at temperature scales and environmental conditions pertaining to biological systems, but more experiments are needed.

Intriguing theoretical hypotheses have been advanced to explain the striking differences between the two Li^+^ isotopes (Fisher, 2015; Weingarten, Doraiswamy, and Fisher, 2016; Zadeh-Haghighi and Simon, 2021), but experimental evidence thus far available has not been sufficient to elucidate the mechanism of such isotope action or to identify the molecular site(s) or target(s) responsible for such downstream Li isotope neurological effects, or to ascertain whether the isotopic difference is caused by the relatively large (∼15%) mass difference between the two isotopes (e.g., as in the kinetic isotope effect or other known mass-independent nuclear quantum effects) or their different nuclear spin (i.e., spin 1 and 3/2 for ^6^Li and ^7^Li, respectively). This is a challenging task since essentially all knowledge regarding Li biochemistry has been established on the basis of studies involving natural Li (^nat^Li) salts, which are mixtures of the two Li isotopes. Compounding the challenge, while Li’s clinical applications are established, the full set of its identified biomolecular targets are neither entirely known nor are all mechanisms fully understood. In the search of the molecular targets for Li^+^ action, many directions have been considered. In this regard, we chose to explore the role of mitochondrial NCLX in its capacity to exchange Ca^2+^ for Li^+^ and as a potential target for Li^+^ isotope differentiation. It has been shown that Li^+^ modulates the activity of the NCLX (Boyman et al., 2013) and, by doing so, could potentially influence Ca^2+^ signaling in the brain. It has been proposed that NCLX has distinct binding sites for Na^+^ and Li^+^ and it was shown that the NCLX_Mj mutant can alternatively bind either one ion of Ca^2+^ or 2 Na^+^/2 Li^+^ ions at different stages of the transport cycle (Giladi et al., 2022). Given the crucial role of Ca^2+^ in the regulation of neuronal processes (Brini et al., 2014), the effects of Li^+^ could occur through modulating Ca^2+^ levels, emphasizing its significance in neurotransmitter release, synaptic plasticity, and overall neuronal function.

In this work, we measured mitochondrial Ca^2+^ efflux in the presence of ^nat^Li^+^, Na^+^, or K^+^, both under functional and inhibited NCLX conditions. Our findings for ^nat^Li and Na salts correlate well with available published data (Ruiz, Alberdi, and Matute, 2014; Rysted et al., 2021). Titration experiments confirm the effects of 0–20 mM Na^+^ or Li^+^ salts on Ca^2+^ efflux rate (Boyman et al., 2013). Interestingly, we observed that the rate of Ca^2+^ efflux drops significantly at increased concentrations of Na^+^ or Li^+^ (at around 30–45 mM) (Supplementary Figures S1A–D). Further, our data demonstrate that the simultaneous presence of Na^+^ and Li^+^ salts results in the enhancement of Ca^2+^ efflux when compared to Li^+^ alone but is similar when only Na^+^ is used. In regard to the latter results, our initial hypothesis was that the interaction of one of the Li^+^ isotopes with NCLX might influence Na^+^-induced Ca^2+^ release differently from that of the other Li^+^ isotope. If occurring, such an effect may be studied in Na^+^/Li^+^ mixtures given the documented distinctions in binding sites for Na^+^ and Li^+^ in NCLX (Giladi et al., 2022). The existence of different binding sites is noteworthy, as these sites may exhibit distinct affinities, potentially influencing the exchanger’s capacity, ionic exchange rate, and/or ionic selectivity. We were unable to detect a significant difference between Ca^2+^ efflux evoked by Na^+^ alone or the efflux caused by the combination of Na^+^ and Li^+^, for a same total concentration of ions, possibly due to the intrinsic limitations in the fluorescence method employed and the small signal induced by Li^+^. However, we observed a difference in Ca^2+^ efflux evoked by Li^+^ acting alone compared to the efflux caused by Li^+^ and Na^+^ together, again for a same ionic concentration. These results suggest a potential cumulative effect of Na^+^ and Li^+^.

While there is a pronounced effect of Na^+^ and Li^+^ on Ca^2+^ transport via NCLX, the role of Li^+^ isotopes had previously not been investigated in this regard. Our studies demonstrate no discernible difference in Ca^2+^ efflux rates triggered by ^nat^Li^+^, ^6^Li^+^, or ^7^Li^+^. We found that CGP-37157 efficiently inhibits Ca^2+^ efflux irrespective of which isotope is used. We also observed no difference between the Ca^2+^ efflux rate triggered by the simultaneous presence of Na^+^ and various concentrations of ^nat^Li^+^, ^6^Li^+^ or ^7^Li^+^. Based on Ca^2+^ fluorescence results, we conclude that NCLX does not differentiate between the two Li^+^ isotopes, suggesting an equivalence in transport function and binding site interactions for ^6^Li^+^ or ^7^Li^+^, or an undetectable small difference between the two isotopes, under our experimental conditions and with the methods used in this work. Having found that NCLX itself does not differentiate between the Li^+^ isotopes, we explored whether there exists an overall differentiation between the two Li^+^ isotopes at the level of the inner mitochondrial membrane, as it is known that Li^+^ can penetrate different biological membranes differently (Sherman, Munsell, and Wong, 1984; Hughes and Birch, 1992; Poet et al., 2023). We used the ICP-MS technique to test whether the two Li^+^ isotopes penetrate mitochondrial membranes differently by measuring the amount of each Li^+^ isotope inside and outside mitochondria.

To establish a baseline for our experiments, we started with Na^+^ and ^nat^Li^+^ simultaneously present and compared these with Li^+^ isotopes. We found that mitochondria uptake Na^+^ preferentially over Li^+^ (^nat^Li^+^/Na^+^ = 0.036 ± 0.050 in matrix compared to ^nat^Li^+^/Na^+^ = 0.448 ± 0.159 in buffer). Here, we need to highlight the point that since experiments with Na^+^ were performed only in the case of functional NCLX, we cannot assume that NCLX is the sole path for Na^+^ entry into mitochondria leading to Na^+^ versus Li+ differentiation. Indeed, it is known that there are other ion exchangers in the mitochondrial inner membrane, such as the Na^+^-H^+^ exchanger (Numata et al., 1998), that might likely preferentially uptake Na^+^ over Li^+^. Therefore, the possibility does exist that Na^+^ is preferentially taken up over Li^+^ due to the availability of multiple pathways for Na^+^ entry.

In the ICP-MS measurements, we found that ^6^Li^+^ is enriched across the mitochondria inner membrane regardless of the presence of CGP-37157 (^7^Li^+^/^6^Li^+^ = 1.122 ± 0.001 in buffer vs. ^7^Li^+^/^6^Li^+^ = 0.681 ± 0.019 in mitochondrial matrix for functional NCLX and ^7^Li^+^/^6^Li^+^ = 1.124 ± 0.001 in buffer vs. ^7^Li^+^/^6^Li^+^ = 0.537 ± 0.029 in mitochondrial with NCLX blocked by CGP-37157). Interestingly, we find a rather large degree of ^7^Li^+^/^6^Li^+^ fractionation in the mitochondrial matrix with functional NCLX, which is even higher when NCLX is inhibited. These results suggest that not only are there other Li^+^ transport pathways in mitochondria that differentiate between the Li^+^ isotopes, but also that NCLX either does not differentiate between the Li^+^ isotopes or has less preference for ^6^Li^+^ compared to other pathways. It would seem likely that Li^+^ isotope differentiation occurs via Na^+^ exchangers, which are present in the mitochondrial inner membrane, since it is well known that Li^+^ can enter Na^+^ channels (Richelson, 1977; Thomsen and Shirley, 2006). We thus conclude that the inner mitochondrial membrane differentiates between Li^+^ isotopes within the precision and accuracy of the ICP-MS method used in the present work, but that this differentiation is not solely due to NCLX, if NCLX fractionates Li isotopes at all. Moreover, and perhaps most importantly, this ^6^Li+ enrichment does not affect the Ca^2+^ release via NCLX, as was shown by our Ca^2+^ fluorescence experiments. From the perspective of our work, these results would suggest that the Li^+^ isotope fractionation exposed through the ICP-MS measurements does not translate into a downstream effect via Ca^2+^ release through the NCLX. This is noteworthy considering that Ca^2+^ is a crucial ion associated with electrochemical neuronal signaling. Thus, our results would suggest that NCLX function cannot be directly related to the Li^+^ isotope effects seen in animal and tissue experiments (Sechzer et al., 1986; Esmaeilpour et al., 2023).

It is of interest to contrast the large Li isotope fractionation we find in the present work with two recent reports on Li isotope fractionation by Deline et al. (2023); Poet et al. (2023). In their work and ours, Li^+^ isotope fractionation by the mitochondrial membrane, using ICP-MS, and Ca^2+^ release, using Ca^2+^-induced fluorescence, were assessed. Deline et al. (2023) reported a Li^+^ isotope effect on Ca^2+^ buffering capacity as signaled by the mitochondria permeability transition pore (mPTP) opening event while they did not find a differential Li^+^ isotope uptake by mitochondria. Conversely, we tested NCLX, and observed that Li^+^ isotope partitioning in mitochondria did not affect Ca^2+^ efflux though NCLX. The experimental design as well as the types of mitochondria (brain and liver) used by Deline et al. (2023) differ from those considered in the present work, and it is therefore not possible at this time to explicitly state the specific reason(s) why the results differ. However, we believe that this question is interesting and worth exploring further using other experimental designs and utilizing more precise methods [e.g., Li purification by ion-exchange chromatography followed by MC-ICP-MS analysis as carried out by Poet et al. (2023)].

Another point of contrast warrants mention. In the recent work of Poet et al. (2023), it was shown that the Na^+^-H^+^ exchanger differentiates between Li^+^ isotopes and transports ^6^Li^+^ at a higher rate than ^7^Li^+^. Similarly to the Poet et al. (2023) results, we see an enrichment of ^6^Li^+^ in mitochondrial matrix compared to an enrichment of ^6^Li^+^ in fibroblast cells. We must point out, however, that our values are not directly comparable — as we utilized different systems, experimental designs and different ways of calibrating the ICP-MS measurements (^nat^Li standard versus ^6^Li/^7^Li 47%/53% mixture). For example, in our case, we are studying isolated heart mitochondria that have large negative membrane potential (−180 mV), while Poet et al. (2023) studied fibroblast cells. We believe that the pathway of Li^+^ isotopes differentiation should be studied more extensively and propose this as a future research project.

Although we observed a discernable Li^+^ isotope differentiation by the mitochondrial membrane, we do not think that these results readily explain those from tissues and animals studies (Sechzer et al., 1986; Esmaeilpour et al., 2023). The lithium isotope effects found by Esmaeilpour et al. (2023) may be due to electrochemical activity which involves ion transport and signaling. As Ca^2+^ signaling is one of the main mechanisms to initiate the neuronal response in the brain, the NCLX, which transports Ca^2+^ in exchange for Na^+^ and Li^+^, may have been considered as a natural route/mechanism where Li^+^ isotopes can affect Ca^2+^ transport and would be a target to differentiate Li^+^ isotopes. However, our Ca^2+^-induced fluorescence measurements reveal no detectable isotope effects on the Ca^2+^ efflux through NCLX, undermining the credibility of NCLX being the origin of the effects seen in brain tissues (Esmaeilpour et al., 2023) and animal behaviour (Sechzer et al., 1986). It would therefore be interesting to investigate alternative mechanisms and pathways to test the effects of Li^+^ isotopes on Ca^2+^ signaling. This may include inositol monophosphatase (IMPase) within the hosphatidylinositol (PI) signaling pathway (Berridge, Downes, and Hanley, 1989), and the effect of Li^+^ on Ca^2+^ channels activity (McCarthy et al., 2016), endoplasmic reticulum (He et al., 2017), and the mitochondrial respiratory chain (Maurer, Schippel, and Volz, 2009). Given Li’s ability to act on multiple targets simultaneously, it might also be worthwhile to explore Ca^2+^ flux as a function of Li^+^ concentration *in vivo* using live cell model systems. It is worth noting that known Li^+^ effects may be attributed to further downstream neuronal or behavioural effects (Pasquali et al., 2010) arising from subtle Li^+^ actions on a variety of molecular targets, which would explain why drastic Li^+^ isotope effects can be observed at the tissue and animal levels but not at the subcellular/cellular levels.

In conclusion, we report a Li^+^ isotope differentiation in heart mouse mitochondria, determined by ICP-MS, that does not affect Ca^2+^ efflux via the sodium/calcium/lithium exchanger (NCLX), measured by Ca^2+^-induced fluorescence. Future research focused on already acknowledged targets for natural isotopic abundance Li may provide promising avenues for exploring Li isotope effects in the realm of neuroscience. However, were such molecular levels of Li isotope action to be convincingly exposed, their consequential downstream effects in neuronal activity will necessitate comprehensive investigations.

## Data Availability

The raw data supporting the conclusion of this article will be made available by the authors, without undue reservation.
