# Supplementary material for: Effects of lithium isotopes on sodium/lithium co-transport and calcium efflux through the sodium/calcium/lithium exchanger in mitochondria
Source: Front Physiol. 2024 Apr 9;15:1354091. doi: 10.3389/fphys.2024.1354091 (PMC11036541; doi:10.3389/fphys.2024.1354091)
Supplement: Supplementary file 1 [file DataSheet1.pdf]

Supplementary Material

These experiments demonstrate that 15-20 mM of Li^+/Na^+ trigger the highest Ca^{2+} efflux rate.

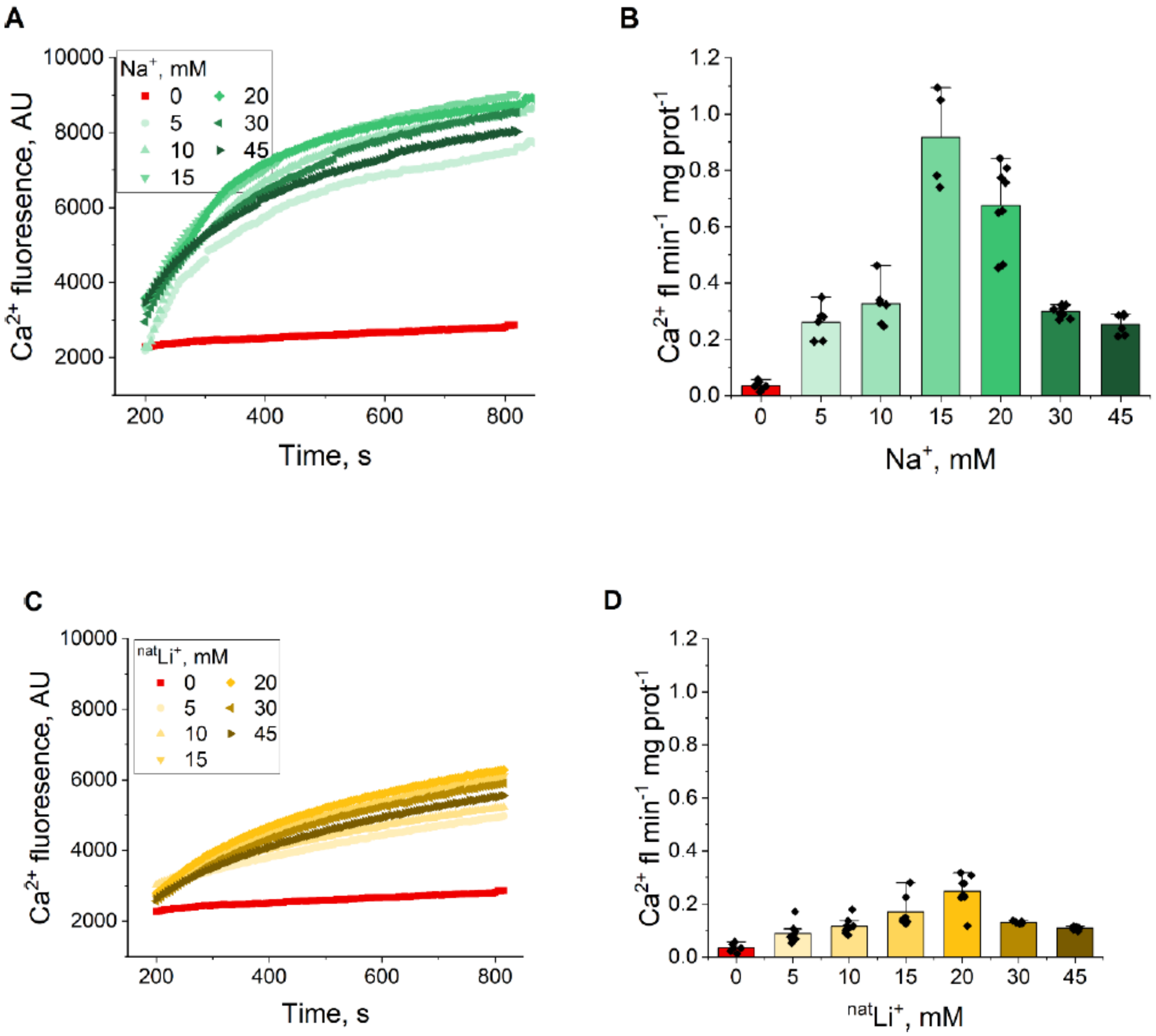

Figures S1A-D. Ca-induced fluorescence as a probe of NCLX activity in the presence of different Na^+/Li^+ concentrations. Representative Ca^{2+} efflux measured from isolated heart mitochondria during a 10 min period where NCLX is functional in the presence of different concentrations of Na^+**(A)** or $^{nat}Li^+$**(C)**. Quantification of the maximal rates of mitochondrial Ca^{2+} efflux induced by different concentrations of Na^+ **(B)** or $^{nat}Li^+$ **(D)** in the continuous presence of Ru360. Data are presented as mean ± SEM, 3-4 biological replicates, each with 2-3 technical replicates.

We observe no difference in Ca^{2+} efflux rate triggered by $^{6}Li^{+}$, $^{7}Li^{+}$, Li^{+} or 45%/55% mix of $^{6}Li^{+}/^{7}Li^{+}$ isotopes.

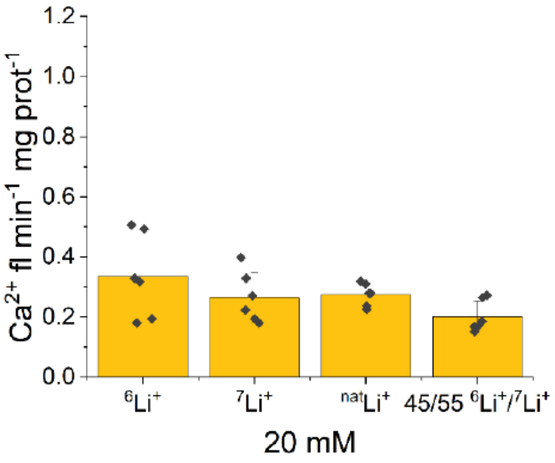

Figure S2. Ca-induced fluorescence in the presence of Li^{+}. Quantification of the maximal rates of mitochondrial Ca^{2+} efflux induced by 20 mM $^{nat}Li^{+}$, $^{6}Li^{+}$, $^{7}Li^{+}$ and 45%/55% Li isotope mixture in the continuous presence of Ru360. Data are presented as mean ± SEM, 3-4 biological replicates, each with 2-3 technical replicates.

	$^{nat}Li^{+}$ [He] ($^{6}Li^{+}$ + $^{7}Li^{+}$)			23 -> $^{23}Na^{+}$ [He]<br>DL = 0.2304 ppb<br>BEC = 6.529 ppb			$^{nat}Li^{+}/Na^{+}$	$^{nat}Li^{+}/Na^{+}$ SD
	Conc. [ppb]	Conc. RSD	Conc. SD	Conc. [ppb]	Conc. RSD	Conc. SD		
Matrix	1.5	7.4	0.1	197.9	0.4	0.9	0.008	0.001
Buffer	345.6	1.4	4.8	1319.1	0.3	3.8	0.262	0.004
Matrix	1.6	8.9	0.1	249.0	0.7	1.6	0.006	0.001
Buffer	536.7	1.8	9.7	908.4	0.9	8.6	0.591	0.012
Matrix	11.6	1.9	0.2	123.5	0.6	0.7	0.094	0.002
Buffer	562.3	1.6	8.9	1147.5	1.1	12.2	0.490	0.009

Table S1. ICP-MS measurements of Na^{+} and $^{nat}Li^{+}$ in mitochondrial matrix and supernatant buffer. Calculation of $^{nat}Li^{+}/Na^{+}$ values. DL = detection limit; BEC = background equivalent concentration; RSD = relative standard deviation; SD = standard deviation.

		6 -> $^{6}Li^{+}$ [He]			7 -> $^{7}Li^{+}$ [He]			$^{7}Li^{+}/^{6}Li^{+}$	$^{7}Li^{+}/^{6}Li^{+}$ SD
		DL = 0.007178 ppb			DL = 0.01425 ppb				
		BEC = 0.01064 ppb			BEC = 0.04273 ppb				
		Conc. [ppb]	Conc. RSD	Conc. SD	Conc. [ppb]	Conc. RSD	Conc. SD		
Without CGP-37157	Matrix	4.1	1.7	0.1	2.7	1.7	0.0	0.663	0.016
	Buffer	150.5	0.8	1.2	168.9	1.3	2.1	1.122	0.017
	Matrix	3.5	1.3	0.0	2.4	1.4	0.0	0.678	0.013
	Buffer	161.0	0.4	0.6	180.5	0.9	1.6	1.121	0.011
	Matrix	8.8	1.0	0.1	6.2	0.7	0.0	0.701	0.008
	Buffer	126.4	1.7	2.2	141.8	1.1	1.6	1.122	0.023
With CGP-37157	Matrix	2.1	2.6	0.1	1.1	1.8	0.0	0.516	0.016
	Buffer	160.6	0.5	0.8	180.3	0.7	1.2	1.123	0.009
	Matrix	4.4	1.3	0.1	2.5	0.9	0.0	0.557	0.009
	Buffer	148.0	0.8	1.2	166.4	0.6	0.9	1.124	0.011

Table S2. ICP-MS measurements of $^{6}Li^{+}$ and $^{7}Li^{+}$ in mitochondrial matrix and supernatant buffer. Calculation of $^{7}Li^{+}/^{6}Li^{+}$ values. DL = detection limit; BEC = background equivalent concentration; RSD = relative standard deviation; SD = standard deviation.
